# Actinomycosis of the Pancreas: A Case Report and Review

**DOI:** 10.4021/gr2010.05.207w

**Published:** 2010-05-20

**Authors:** Amalanshu Jha, Anusak Yiengpruksawan, Arthur A. Christiano, Neeti Jha, Prajna Latika

**Affiliations:** a Department of Surgery, Bronx-Lebanon Hospital Center, Bronx, New York, USA; b Surgical Oncology, Minimal Invasive and Robotic Surgery, The Valley Hospital, Ridgewood, New Jersey, USA; c Department of Pathology, The Valley Hospital, Ridgewood, New Jersey, USA; d Department of Internal Medicine, Lincoln Medical and Mental Health Center, Bronx, New York, USA; e Department of Internal Medicine, Bronx-Lebanon Hospital Center, Bronx, New York, USA

**Keywords:** Pancreas, Actinomycosis, Intraductal papillary mucinous tumor (IPMN)

## Abstract

*Actinomyces* is a normal commensal of the upper aerodigestive tract, colon and female reproductive tract. It can give rise to invasive disease in case of any breach in mucosal integrity, as well as, in patients with immunosuppression. Rarely, actinomycosis can involve the pancreas especially after episodes of pancreatitis or in post operative patients. We observed a case of actinomycosis affecting recurrent intraductal papillary mucinous neoplasm (IPMN) of pancreatic remnant, 5 years after a Whipple's procedure. Our patient, a 66 years old male with a history of Whipple's procedure for IPMN of pancreatic uncinate process, presented with repeated episodes of acute pancreatitis. Repeated radiological investigations (CT, MRI and EUS) revealed resolving pancreatitis with recurrent IPMN of the pancreatic tail. The patient underwent laparobotic assisted resection of the remnant pancreas and spleen 3 months later. Intraoperatively, in addition to the recurrent IPMN of pancreatic tail, we found a dense peripancreatic desmoplastic reaction with areas of thick yellow pus pockets in the remnant pancreatic body. Bacteriology and histopathology revealed it as a recurrent IPMN associated with actinomycosis of pancreas with chronic xanthogranulomatous changes. We conclude that actinomycosis of the pancreas is a rare entity with only 5 cases reported in English literature to the best of our knowledge. If diagnosed preoperatively, early institution of antibiotics can improve the surgical outcome. Fortunately, after diagnosis, we were able to start antibiotics in early postoperative period with successful outcome.

## Introduction

*Actinomyces* is a normal commensal of the upper aerodigestive tract, colon and female reproductive tract. It can give rise to invasive disease after a breach of mucosal integrity, as well as, in patients with immunosuppression [1-6].

It causes an indolent, fibrosing infection of any organ, with formation of sinuses discharging pus with ‘sulfur granules’. It has been shown to form internal fistulae, mucosal/skin ulcers [7] and occasionally an inflammatory mass which can mimic a malignancy [8, 9].

Actinomycotic involvement of the pancreas is extremely rare. We report a case of actinomycosis of the pancreatic remnant having recurrent intraductal papillary mucinous neoplasm (IPMN). Our patient had pancreaticoduodenectomy 5 years ago for IPMN of the pancreas. Subsequently, he had several episodes of pancreatitis before being diagnosed with recurrent IPMN of pancreas. Actinomycosis was reported in the resected pancreas and the splenic hilum.

## Case Report

A 66 years old man presented with recurrent epigastric pain. Computed tomography (CT) showed a 3 cm cystic lesion in the uncinate process of the pancreas. Endoscopic ultrasonography (EUS), further, revealed a 2.6 cm x 1.6 cm cyst in the uncinate process with an irregular wall and papillary growth.

He underwent a Whipple's procedure at a different hospital. The cyst was found to be an IPMN. His postoperative course was stormy with pancreatitis of the pancreatic remnant, pulmonary embolism necessitating an IVC filter placement and prolonged hospitalization (2 months).

**Figure 1:**
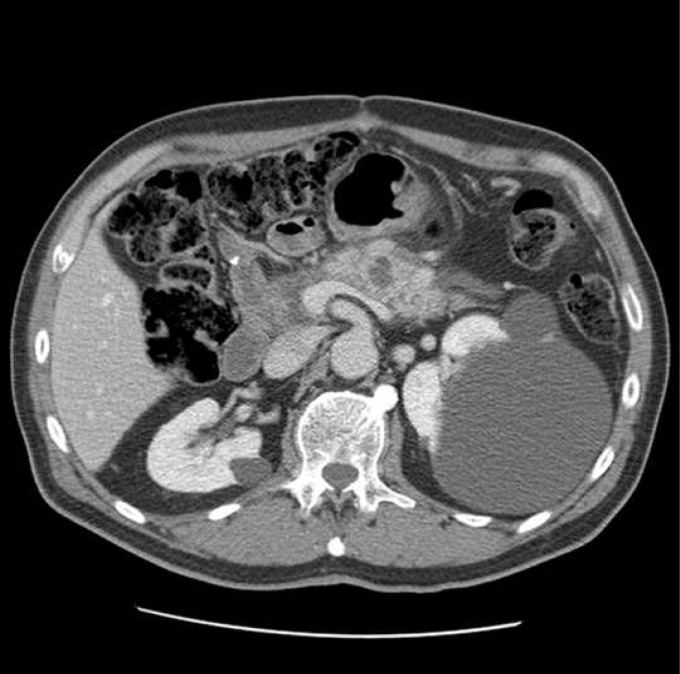
Acute pancreatitis with fluid collection.

Five years later he presented with symptoms of acute pancreatitis. A CT scan showed pancreatitis with a 15 mm acute fluid collection within the pancreatic remnant (Fig. 1).

**Figure 2:**
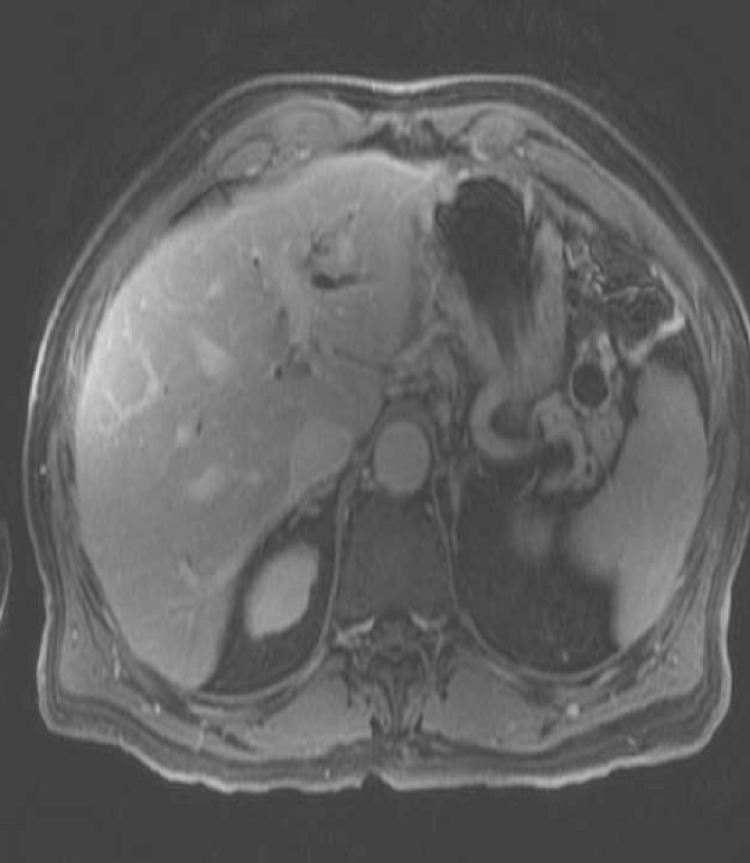
Recurrent IPMN cyst of pancreatic tail.

**Figure 3:**
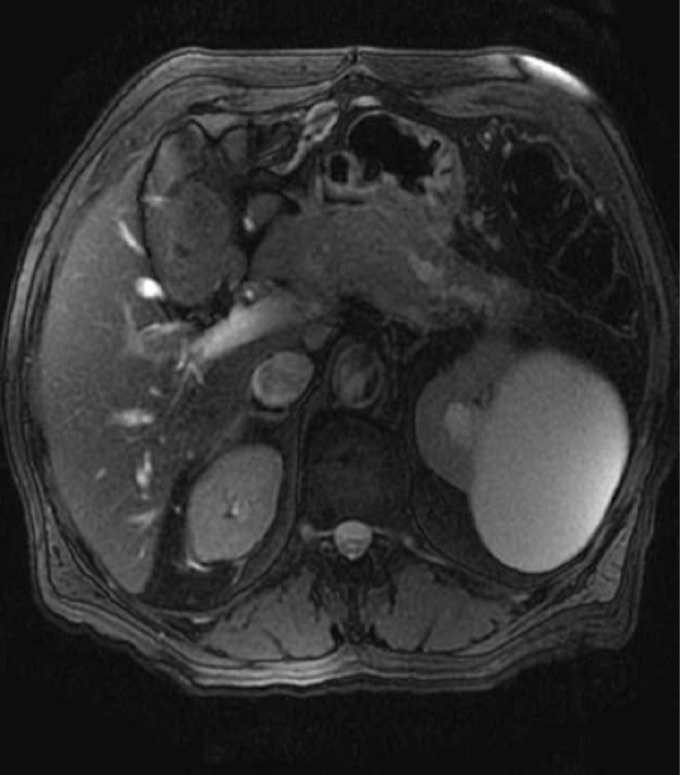
Pancreatic duct stenosis with distal dilatation.

The patient had an uneventful recovery from pancreatitis. He continued to have episodes of epigastric pain, malaise and weight loss over next 5 months. Follow up Magnetic resonance cholangiopancreatography (MRCP) two months after pancreatitis showed resolution of acute fluid collection. It also revealed a new cystic lesion in the pancreatic tail (Fig. 2) with stenosis of the main pancreatic duct and distal dilatation (Fig. 3). Further evaluation by EUS showed a 22 mm x 24 mm cyst in the pancreatic tail.

**Figure 4:**
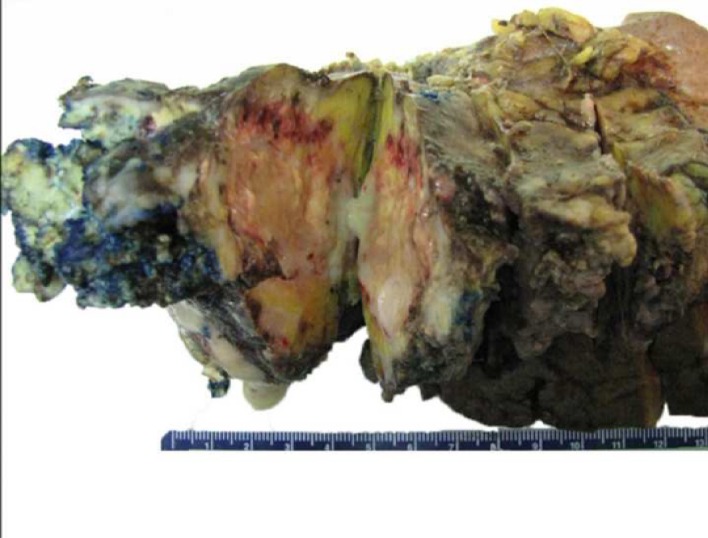
Gross specimen of resected remnant pancreas with spleen.

The patient underwent laparobotic assisted pancreaticosplenectomy 3 months later. Intraoperative findings included a cyst (recurrent IPMN on frozen section) in the pancreatic tail. Additionally, there was extensive fibrosis around the pancreas with adhesions to the surrounding structures including the stomach and the splenic flexure of colon. Several yellow pus pockets were found in the pancreatic tail extending to the splenic hilum (Fig. 4).

**Figure 5:**
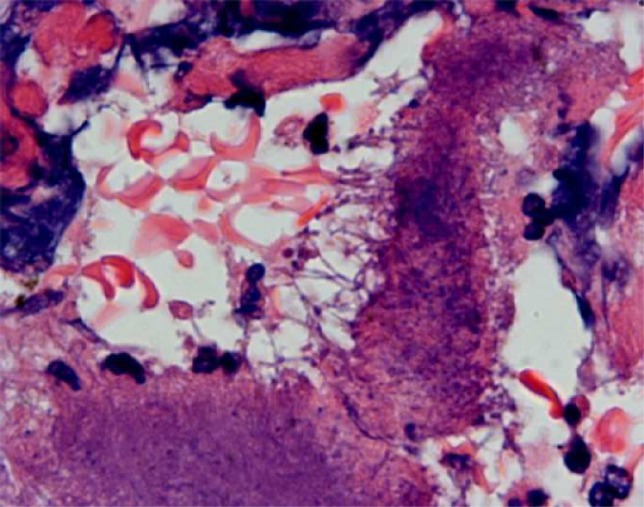
Branching bacterial filaments at the edge of sulfur granule. Note surrounding inflammation.

Histopathology revealed recurrent IPMN with colonies of actinomyces in the pancreatic tissue in a background of inflammatory infiltrate and foamy histiocytes - suggestive of chronic xanthogranulomatous pancreatitis (Fig. 5).

The patient was treated with intravenous penicillin for 4 to 6 weeks followed by oral amoxicillin for further 6 to 8 months. The patient is healthy at 2 years of follow-up.

## Discussion

### Etiology and pathogenesis

Actinomycosis is a rare opportunistic disease usually caused by the species *Actinomyces israelii*. Other species in the genus *Actinomyces* which may cause human infections include: *A. odontolyticus, A. naeslundii, A. meyeri, A. gerencseriae* and *A. viscosus* [10]. These organisms were once believed to be fungi due to their branching filamentous morphology. They lack a nuclear membrane; their cell walls lack chitin and are affected by penicillin but not by antifungal agents [11]. Hence, they are now considered to be higher forms of prokaryotic bacteria.

They are gram positive filamentous bacteria. They are typically anaerobic or microaerophilic, hence surviving as commensal in oral cavity, gastrointestinal tract and female genital tract. In fact, oral colonization has been shown to occur in 97% of infants by 2 years of age [12]. No known natural source or natural habitat is known besides human beings and animals.

They can acquire pathogenicity after invasion of tissues. They are known to enter tissues with any mucosal breach due to dental procedures, oral surgery [3], after inflammatory diseases of gastrointestinal tract including diverticulitis, appendicitis and intestinal perforation, and post surgery [4-6] or long standing intrauterine contraceptive devices [2]. Foreign bodies such as fish bone [1], IUCD's [2] have been reported to facilitate infection.

Infection is usually chronic and slowly progressive. Due to its infiltrating nature, it causes a dense desmoplastic reaction in the surrounding tissue. It slowly grows by contiguous spread ignoring anatomical planes. With further growth, the center of the infection site liquefies to become purulent material.

Indurated lesions are more apparent in abdominal or cervicofacial locations as compared with pulmonary or central nervous system (CNS) lesions. Sinus tracts might form to the skin or surrounding viscera due to its infiltrative nature.

Microscopically, ‘sulfur granules’ are the hallmark of the disease. They consist of a conglomeration of filamentous bacteria. The filaments are better visualized at the margins of these granules. The surrounding tissues show dense inflammatory infiltrates of neutrophils, lymphocytes and foamy macrophages.

### Clinical presentation

Actinomycosis can involve any tissue of the body, but clinical patterns can be classified broadly into 6 categories in the order of frequency: Oro-cervicofacial (55%) [4], abdominopelvic (20%) [4, 11], thoracic (15%) [13], rarely CNS, musculoskeletal and disseminated actinomycosis.

In oral and cervicofacial region, actinomycosis can present as a soft tissue swelling, abscess, sinus, mass [4] or an ulcer [7]. Although any part of oral cavity or neck can be involved, the classic location is described at the angle of mandible [14].

In the thorax, the disease commonly presents as a pulmonary mass with ‘air bronchogram’ (patent bronchus in a mass, also called ‘open bronchus sign’) [15], pneumonia, empyema or cavitations (usually multiple) [15]. Mediastinal disease, although rare, commonly involves pericardium [16].

Rarely, the CNS is involved. Brain abscess is the most common presentation with CT showing ring-enhancing lesions.

Abdominal actinomycosis commonly presents as a slowly growing mass mimicking malignancy or as an abscess. It can involve any site in the abdominal cavity but it is more frequent in the ileocecal region [17]. Other areas reported to be involved include liver [18], gallbladder [19], pelvis (perianal and perirectal disease) and the retroperitoneum including ureters and kidneys [20].

Pancreatic involvement is extremely uncommon. It has been reported only 5 times in the past to the best of our knowledge (Table 1). The earliest report was by Parsons et al [21] in 1929 of a fatal case of pancreatic actinomycosis, initially suspected as pancreatic necrosis. In all the other case reports, the initial suspicion was that of pancreatic malignancy due to their presentation as a pancreatic mass. Two patients had chronic stent associated pancreatic actinomycosis [18] and three were linked to prior surgery [8, 9, 22]. Chronic pancreatitis was associated with actinomycosis in 3 patients [8, 18], while prior pancreatic surgery was present in 2 patients [8, 9] and one patient had both prior surgery and pancreatitis [8] as in our patient.

**Table 1:** Reported Cases of Pancreatic Actinomycosis in Literature

Author	Presentation	Initial Suspicion	Believed Cause	Part Involved	Diagnosed by	Treatment	Alive
Parson [21]	Obstipation, Sepsis, Peritonism	Pancreatic necrosis	Pancreatitis	Whole pancreas	Surgery – Final diagnosis by bacteriology of wound draining sulfur granules	Sodium iodide, Copper sulfate, Colloidal copper	Died 3 months later
Ma [8]	Abdominal pain, CT showing 3 cm mass in pancreatic head, 9 years post PJ^a^ for chronic pancreatitis	Malignancy of pancreatic head	Chronic alcoholic pancreatitis Prior PJ^a^ for pain (9 yrs ago)	Head	EUS guided FNA	1 year course of Amoxicillin	Yes
Harsch [18]	Pain, fever, night sweats, CT Pancreatic head lesion and hepatic lesion	Malignancy initially	Chronic pancreatitis, Chronic PD^b^ stenting	Head	USG guided percutaneous aspiration of liver lesion	Penicillin G – Clindamycin	Yes
Harsch [18] (2nd Patient)	Pain, fever, sepsis with pancreatic head lesion on CT	Malignancy initially,	Chronic pancreatitis Chronic PD^b^ stenting	Head	Stent bacteriology, percutaneous CT guided liver abscess culture	Imipenem Clindamycin, vancomycin	Yes
Jun-Te Hsu [9]	2 years after PD^c^ for periampullary carcinoma, mass at PJ^a^ site	Recurrent periamp^d^ carcinoma	Prior Whipple’s procedure	PJ^a^ site	Surgery – resection of anastomotic mass and reconstruction of PJ^a^	Penicillin	Yes
Halevy [22]	Right hypochondrial pain, fever, weight loss, palpable mass RUQ	Pancreatic carcinoma	Reflux to PD^b^ / ?post appendicitis contiguous spread	Head	Laparotomy	Penicillin 600 mg & TMP-SMX^e^	Yes
Our patient	Epigastric pain, recurrent IPMN in tail of pancreas, 5 years after Whipple	Recurrent IPMN	Prior Whipple’s procedure, Chronic pancreatitis	Tail and splenic hilum	Surgery, distal pancreatectomy and splenectomy	Penicillin – Amoxicillin	Yes

^a^pancreaticojejunostomy, ^b^pancreatic duct, ^c^pancreaticoduodenectomy, ^d^periampullary, ^e^Trimethoprim Suflamethoxazole

We believe that our patient had the seeding of pancreas by actinomyces by repeated episodes of pancreatitis aided by reflux of gastrointestinal contents via the anastomosis of remnant pancreatic duct to jejunum.

In a symptomatic patient who had a prior surgery and also who had recurrent episodes of pancreatitis, it is not unreasonable to consider actinomycosis of the pancreas in the differential diagnosis. A CT or EUS guided FNA of inflammatory collection around pancreas may yield the diagnosis of actinomycosis [8] which can then be successfully treated by antibiotics. This may improve the surgical outcome in elective or semi-emergent cases.
